# Valedictory Address

**Published:** 1866-12

**Authors:** H. J. M’Kellops


### VALEDICTORY ADDRESS.

BY H. J. M'KELLOPS, D. D. S.

Retiring President of the Missouri Dental Association.

Gentlemen:—In retiring from the honorable position to which I have been elevated by my colleagues of the Missouri Dental Association, I feel it a pleasing duty to address you a few words in reference to the position, advancement and progress of Dentistry in its relation to the social scale.

In an address of this kind, I do not propose to make it an opportunity of saying anything new, but to offer some general remarks upon the relation that Dentistry holds to society at large, in order that we may the more clearly appreciate the character and extent of that useful mission in which we have all embarked.

A few years ago, and within the memory of many of us, the science of Dentistry was wholly unknown; and it occupied neither position nor status in the world of medical progress, and was considered a mere appendage of questionable value in the life of the professional man. Since then, step by step, it has advanced, gathering strength by every movement, until now it justly claims a position ennobled by the practical applications and vivifying influences of those who have labored for its advancement.

Much has been done and much more remains to be accomplished, before our art shall have reached that degree of perfection which awaits patient and progressive research in the unexplored fields of experimental philosophy. To discharge conscientiously the implied contract entered into with our patients, to effect the most perfect specimen of our handicraft, is nothing more than is expected in *every* commercial transaction. If nothing more than this were done, Dentistry would hardly rise above the level of the numerous trades and callings of life. But it has gained an honorable position in the profession of medicine and surgery, by the aid of those illustrious men who have devoted energy, industry, and perseverance to its development as a science.

It should be our aim and ambition, then, to perfect the possession of that inheritance which the genius, the philanthropy and the devoted industry of others have accumulated, and not content ourselves with merely performing a piece of artistic workmanship.

It should be a special object of the members of this society to still further augment the sphere of its usefulness, and by acquiring an intimate knowledge of physical and biological science which truly belongs to the domain of odontologic art.

It is the study and application of the laws and principles that govern matter, and the influences they exert upon the vital organism, that marks the advancement of medicine as a science, and which equally pertains to the department of dentistry, a distinctive branch of the profession. If we begin to study the phenomena and laws of life in man, we shall soon be irresistibly attracted to extend our observations to the successive orders of creation, and so on down to the lowest type of animated matter. As we advance in this study, the beauty, harmony, and correlation of organic and inorganic forces open up to our vision. We see the adaptation of those mysterious principles in the vegetable and mineral kingdoms, so potent in the consulting room and laboratory, and the knowledge of each plant or mineral becomes more perfectly understood by the multiplying beams of intelligence reflected at every step.

As lovers of our art, then, let it be our chief pleasure to elevate it, year by year, until it shall have occupied a position prominent among the most dignified specialties in the medical profession. This is to be effected, not merely by the aid of mechanical skill in remedying deformity, and relieving suffering, but in applying the principles of biology and physical science to the prevention of disease, the preservation of the teeth, rather than their destruction by mechanical agents.

Our object should be to comprehend the effort that nature is making in combatting the adverse influences under which she is laboring. Let us seek to aid her in the conflict, observing and following her laws, that we may minister to her short-comings. If we deviate from this path; if, losing sight of the beacon of observation, we chase the *ignis fatuus* of speculation, we shall just so surely miss our aim, and fail to benefit our patient. He who would minister to nature must learn to interpret her. It is by the exercise of this prerogative that the educated and accomplished Dentist excels the mere mechanic, whose chief merit consists not in preserving the teeth, but in making close imitations.

Besides the channels of investigation just pointed out, the successful practitioner of dentistry is required, not only to understand the anatomical construction of the teeth, and whatever pertains to their growth, position and relation to other parts of the mouth, but he should continue his research into what constitutes the domain of Physiology—a research which carries its investigations in quest of analogies and illustrations into the widest latitudes of the animal and vegetable kingdoms, as well as into the most minute inspection of the structures, the functions, and the attributes of their several productions.

In the practice of Dental Surgery a profound knowledge of the anatomy of all parts contained within the buccal cavity, is absolutely necessary to success. Anatomy is also the foundation of the diagnosis of diseases that fall under the observation and treatment of the dentist. It demonstrates the normal condition of structures in health, and supplies the means of comparison in the study of dental pathology. Neither anatomy, nor any power of ocular investigation, however accurate it may be conducted can enable the dentist to obtain a correct knowledge of the constituent formations of the teeth and modifications they undergo in their alveoli. These things must be learned through the aid of a microscope, and this adds another auxiliary branch of investigation to the perfection of Dentistry as a science.

The art of Dentistry, then, comprehends a perfect knowledge of Anatomy, Physiology, Pathology, the continual use of the microscope, and an understanding of the laws and principles that pertain to biological and physical science. Let each one of us learn as much of them as we can, always bearing in mind the great object for which they are studied, and never neglecting the purpose to which they are to be applied. We should remember that dentistry, as a science, was founded and raised upon the natural sciences. All that it is beyond a mere empirical art, it owes to its dependence upon, and its association with these. It would be difficult to find any one in our ranks who has attained great eminence in the dental art, or who, by his writings and precepts, has advanced our knowledge in the treatment of diseased teeth, or other parts within the mouth, who did not lay the foundation of his successes in the distinction which he earned by his researches in the natural sciences.

We have met here to-day to furnish our quota towards the advancement of our profession, and its elevation among the liberal and learned professions. It is the *animus* that stimulates our energies, and the energies of all those who truly love their calling. We come here to labor for its continued prosperity, and to communicate whatever of interest we may have learned since the last session of this society. We come here imbued with the spirit of progress, and to lay before our professional brethren our views and observations on those subjects most calculated to improve and instruct them, and benefit our patrons. Let all participate in the deliberations of the session, and whatever of surgical or mechanical interest may have been learned during the past year, let it be given to the society, for the honor of the profession and for the benefit of suffering humanity. If any improvement in Mechanical Dentistry has been made, or new operations devised, let the inventor be induced to spread it upon the records of the society, and receive the meed of praise due him in contributing his share towards the elevation of his chosen profession. The selfish and empirical fashion of secreting improvements, of whatever nature, pertaining to our calling, for the sake of individual aggrandizement, is unworthy the fellowship of honorable men, and must, sooner or later, recoil in shame and odium upon its author. It is to be hoped that such members of our society are, like " angels visits, few and far between." Let me invoke the profession in Missouri, to enter their protest against acts so derogatory to our high calling as benefactors of the human family and let me impress upon them the necessity of instructing their delegates to the National Convention, to guard well the portals of the profession, and shut out such harpies from all deliberation and association in such assemblage.

Finally, gentlemen, let me call your attention to the importance of aiding, with your money and influence, our only educational institution in the West. I refer to the Ohio Dental College, which is doing a good work in extending the usefulness of the profession, and advancing its importance in the social scale.

In conclusion, permit me to thank you for the honor conferred upon me, in selecting me as your presiding officer for the past year, and the zeal and interest you have manifested in keeping our science "pure and spotless in the world."

